# Data on the association of serum glypican-4 with future major adverse cardiovascular events and mortality in patients undergoing coronary angiography

**DOI:** 10.1016/j.dib.2022.108142

**Published:** 2022-04-09

**Authors:** Axel Muendlein, Eva Maria Brandtner, Andreas Leiherer, Kathrin Geiger, Christine Heinzle, Stella Gaenger, Peter Fraunberger, Arthur Mader, Christoph H. Saely, Heinz Drexel

**Affiliations:** a Vorarlberg Institute for Vascular Investigation and Treatment (VIVIT), Feldkirch, Austria; b Medical Central Laboratories, Feldkirch, Austria; c Private University of the Principality of Liechtenstein, Triesen, Liechtenstein; d Department of Internal Medicine I, Academic Teaching Hospital Feldkirch, Feldkirch, Austria; e Department of Medicine and Cardiology, Academic Teaching Hospital Bregenz, Bregenz, Austria; f Drexel University College of Medicine, Philadelphia, PA, USA

**Keywords:** Mortality, MACE, Cardiovascular risk factors, Biomarker, Glypican-4, GPC4

## Abstract

This data article is associated to the research article titled ‘Serum glypican-4 is a marker of future vascular risk and mortality in coronary angiography patients' (Muendlein et al., 2022). The present article provides additional prospective data on the association of serum glypican-4 (GPC4) with the incidence of future major adverse cardiovascular events (MACE), vascular mortality, and overall mortality in a cohort of 760 coronary angiography patients.

Serum GPC4 levels significantly differed between patients with or without an event during follow up. The results were confirmed in subgroup analyses with respect to age, sex, type 2 diabetes mellitus, obesity, the presence of significant coronary stenoses, and renal function, as well as medical treatment. That said, an interaction term between GPC4 and impaired renal function and between GPC4 and the use of beta blockers on the incidence of future fatal events reached statistical significance. In addition, C-statistics were performed showing an additional predictive value of categorized GPC4 to a basic risk model including traditional risk factors for overall mortality.

## Specifications Table

**Table:** 

Subject	Cardiology and Cardiovascular Medicine
Specific subject area	Associations of serum glypican-4 with major adverse cardiovascular events, vascular mortality, and overall mortality taking into account subgroup analyses with respect to covariates.
Type of data	Table Figure
How the data were acquired	Consecutive patients referred to elective coronary angiography were enrolled. Laboratory measurements were performed on sera of venous blood samples collected after a 12 h overnight fasting period. Standard blood parameters were measured in fresh serum samples by routine laboratory methods; GPC4 levels were measured from remaining serum samples stored at −80 °C with a commercially available ELISA kit. During follow up the incidence of major adverse cardiovascular events, vascular mortality, and all-cause mortality were recorded. SPSS 26.0 (SPSS, Inc., Chicago, IL) and R statistical software v. 3.2.3 (http://www.r-project.org) were used for statistical analyses.
Data format	Raw Analyzed Filtered
Description of data collection	Only patients with available serum samples for laboratory analyses and follow-up data were included in the present study. GPC4 levels were measured in serum samples stored at −80 °C. Date and causes of death were obtained from a national survey (Statistik Austria, Vienna, Austria) or from hospital records.
Data source location	• Institution: Vorarlberg Institute for Vascular Investigation and Treatment (VIVIT) • City/Town/Region: Feldkirch, Vorarlberg • Country: Austria
Data accessibility	***All data referred to in your data article must be made publicly available prior to publication.*** Repository name: Mendeley Data Data identification number: 10.17632/cfd9z7ry46.1 Direct URL to data: https://data.mendeley.com/datasets/cfd9z7ry46/1 Data were fully anonymised and therefore no access control is needed
Related research article	A. Muendlein, E. M. Brandtner, A. Leiherer, K. Geiger, C. Heinzle, S. Gaenger, P. Fraunberger, A. Mader, C. H. Saely, H. Drexel, Serum glypican-4 is a marker of future vascular risk and mortality in coronary angiography patients, Atherosclerosis, 2022; 345:33–38. doi:10.1016/j.atherosclerosis.2022.02.015.

## Value of the Data

• The association between serum GPC4 and the risk of future cardiovascular events or death has been unknown, so far. Our data provide new information on the impact of serum GPC4 on the incidence of major adverse cardiovascular events, vascular mortality, and all-cause mortality in cardiovascular risk patients.
• The data benefit researchers as well as health care professionals in the fields of cardiology, nephrology and general internal medicine.
• Our data should stimulate the development of study protocols to gain further insight into the association of GPC4 and other markers of glycocalyx damage with the outcome of cardiovascular risk patients, including prospective investigations and interventional studies.

## 1 Data Description

Table 1 shows the GPC4 levels of patients with or without major adverse cardiovascular events (MACE), vascular mortality, and all-cause mortality occurred during the follow-up period. GPC4 levels are given as median [interquartile range]. Statistical significance was defined as a *p*-value of < 0.05.

**Table 1:** GPC4 levels of patients with or without an event during follow up.

	Event during follow up	N°	GPC4 level (ng/ml)	*p*-value
MACE	No	623	5.5 [4.6–7.0]	< 0.001
	Yes	137	6.3 [5.0–8.6]	

Vascular mortality	No	693	5.6 [4.6–7.0]	< 0.001
	Yes	67	7.3 [5.5–8.9]	

All-cause mortality	No	615	5.5 [4.6–6.7]	< 0.001
	Yes	145	7.3 [5.5–8.9]	

*P*-values were obtained by the Mann–Whitney U test. Abbreviations: MACE, major adverse cardiac event.

Table 2 shows results from C-statistics assessing the utility of GPC4 in risk prediction models for adverse outcomes. A basic model including sex, age, type 2 diabetes mellitus (T2DM), smoking, body mass index, total cholesterol, C-reactive protein, blood pressure, eGFR, and angiographically determined significant coronary artery disease (CAD) was built as a linear predictor score after Cox regression. The basic model was compared with a model including variables of the basic model and additionally serum GPC4. Table 2 shows the area under the curves, Harrell's C and Somers' D for both models as well as the results from DeLong's test indicating an additional predictive value of GPC4 to the basic prediction model for overall mortality. No additional value of GPC4 to a basic risk model was found for the prediction of MACE or vascular mortality.

**Table 2:** Comparison of the area under the curve of prediction models for the incidence of MACE, vascular mortality, and all-cause mortality.

	Model	AUC (95% CI)	Harrell's C	Somers' D	*p*-value	Z for DeLong's test
MACE	Basic	0.701 (0.653–0.749)	0.686	0.371	–	
	Basic+GPC4	0.706 (0.658–0.753)	0.693	0.385	0.468	−0.726

Vascular mortality	Basic	0.807 (0.757–0.856)	0.805	0.610	–	
	Basic+GPC4	0.809 (0.762–0.856)	0.815	0.630	0.760	−0.306

All-cause mortality	Basic	0.800 (0.761–839)	0.775	0.551	–	
	Basic+GPC4	0.823 (0.786–0.859)	0.794	0.588	0.027	−2.215

Models were built as linear predictor scores after Cox regression. The basic model comprises sex, age, T2DM, smoking, BMI, total cholesterol, C-reactive protein, blood pressure, eGFR, and angiographically determined significant CAD. The basic model was compared with a model including variables of the basic model and additionally serum glypican-4. AUCs were compared according to DeLong's test; respective *p-value*s are given for the comparison with the basic model. Abbreviations: MACE, major adverse cardiac event, AUC, area under the curve; CI, confidence interval; CKD, chronic kidney disease, GPC4, glypican-4.

**Fig. 1:**
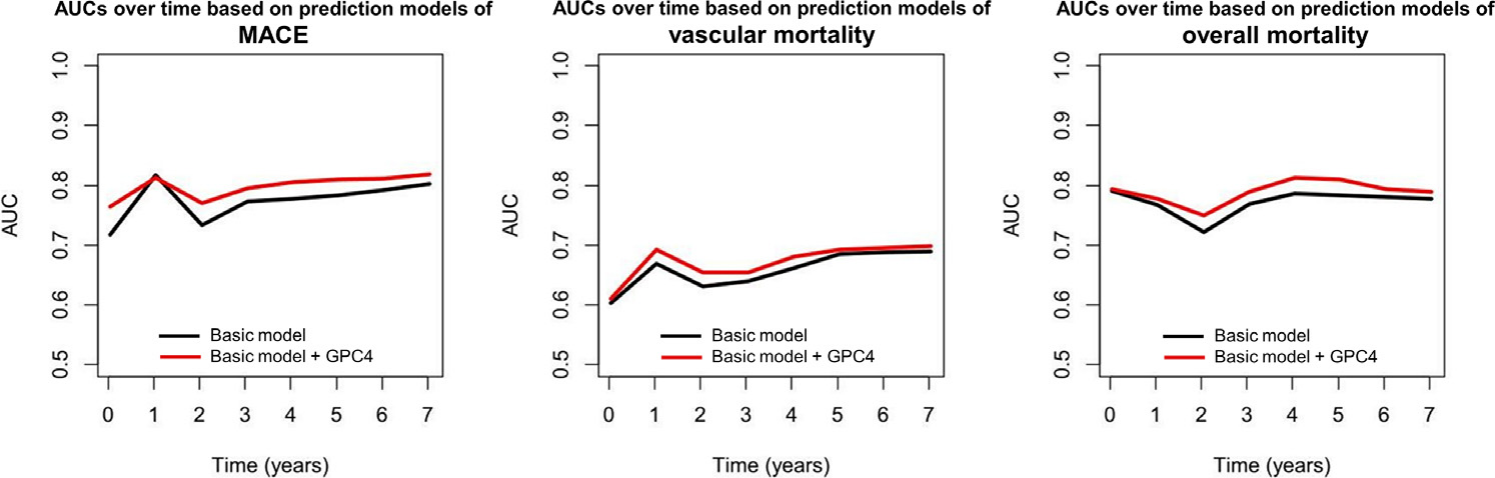
Time-dependent ROC analysis. Area under the curve (AUC) over time based on prediction models of major adverse cardiovascular events (MACE), vascular mortality, and overall mortality. The plots represent the AUCs for a basic prediction model (black line) including age, sex, body mass index, blood pressure, type 2 diabetes mellitus, smoking, total cholesterol, estimated glomerular filtration rate, C-reactive protein, and angiographically determined significant coronary artery disease and the basic prediction model additionally including GPC4 (red line) during the full follow up time.

Fig. 1 shows time-dependent area under the curves of GPC4 for MACE, vascular mortality, and all-cause mortality occurred during the follow up period.

Table 3 presents results from Cox proportional hazards regression analyses in subgroups regarding the impact of serum GPC4 on the incidence of MACE. Subgroups were stratified by sex, median age, obesity, T2DM, kidney function, and the presence of significant CAD at angiography as well as the use of statins, beta blockers, and angiotensin converting enzyme inhibitors. Table 4 shows the association of serum GPC4 with vascular mortality and Table 5 shows the association of serum GPC4 with overall mortality in respective subgroups. High serum GPC4 was consistently associated with adverse outcomes across subgroups. In this regard, an interaction term between GPC4 and decreased kidney function as well as between GPC4 and the use of beta blockers on the incidence of fatal events was statistically significant.

**Table 3:** Association between serum GPC4 and major adverse cardiac events in subgroup analyses.

	N° of controls/events	HR [95%CI]	*p-value*	*P*_interaction_
Age < 67 years	328/46	1.25 [0.98–1.58]	0.068	0.143
Age ≥ 67 years	295/91	1.56 [1.30–1.86]	< 0.001	
Male	385/95	1.38 [1.12–1.59]	< 0.001	0.142
Female	238/42	1.70 [1.33–2.18]	< 0.001	
BMI < 30 kg/m^2^	456/101	1.44 [1.26–1.65]	< 0.001	0.960
BMI ≥ 30 kg/m^2^	167/36	1.45 [1.09–1.93]	0.011	
eGFR ≥ 60 ml/min/1.73 m^2^	575/104	1.32 [1.06–1.63]	0.013	0.603
eGFR < 60 ml/min/1.73 m^2^	48/33	1.21 [0.97–1.50]	0.086	
No T2DM	475/82	1.39 [1.18–1.64]	< 0.001	0.714
T2DM	147/54	1.46 [1.19–1.80]	< 0.001	
No significant CAD	302/40	1.51 [1.26–1.80]	< 0.001	0.752
Significant CAD	321/97	1.46 [1.22–1.74]	< 0.001	
No statin treatment	318/62	1.46 [1.25–1.71]	< 0.001	0.973
Statin treatment	305/75	1.46 [1.19–1.78]	< 0.001	
No beta blocker treatment	282/53	1.44 [1.22–1.70]	< 0.001	0.612
Beta blocker treatment	341/84	1.52 [1.24–1.87]	< 0.001	
No ACE inhibitor treatment	443/88	1.46 [1.27–1.68]	< 0.001	0.817
ACE inhibitor treatment	180/49	1.40 [1.07–1.84]	0.014	

The results were obtained from crude Cox proportional hazards regression analyses and are presented as hazard ratios (HR) and 95% confidence intervals (CI) for one standard deviation change of serum GPC4. Abbreviations: BMI, body mass index; eGFR, estimated glomerular filtration rate; T2DM, type 2 diabetes mellitus; CAD, coronary artery disease; ACE, angiotensin converting enzyme.

**Table 4:** Association between serum GPC4 and vascular mortality in subgroup analyses.

	N° of controls/events	HR [95%CI]	*p-value*	*P*_interaction_
Age < 67 years	361/13	1.37 [0.94–1.99]	0.104	0.604
Age ≥ 67 years	332/54	1.53 [1.23–1.91]	< 0.001	
Male	429/51	1.54 [1.31–1.81]	< 0.001	0.885
Female	264/16	1.49 [1.00–2.24]	0.051	
BMI < 30 kg/m^2^	507/50	1.61 [1.38–1.88]	< 0.001	0.117
BMI ≥ 30 kg/m^2^	186/17	1.08 [0.67–1.73]	0.755	
eGFR ≥ 60 ml/min/1.73 m^2^	631/48	1.60 [1.20–2.12]	0.001	0.090
eGFR < 60 ml/min/1.73 m^2^	62/19	1.10 [0.81–1.50]	0.543	
No T2DM	525/32	1.60 [1.30–1.97]	< 0.001	0.820
T2DM	167/34	1.31 [1.00–1.70]	0.047	
No significant CAD	321/21	1.52 [1.19–1.93]	0.001	0.754
Significant CAD	372/46	1.61 [1.28–2.03]	< 0.001	
No statin treatment	348/32	1.57 [1.30–1.90]	< 0.001	0.727
Statin treatment	345/35	1.48 [1.12–1.96]	0.007	
No beta blocker treatment	305/30	1.40 [1.12–1.76]	0.003	0.120
Beta blocker treatment	388/37	1.85 [1.40–2.43]	< 0.001	
No ACE inhibitor treatment	490/41	1.47 [1.21–1.79]	< 0.001	0.332
ACE inhibitor treatment	203/26	1.78 [1.29–2.47]	0.001	

The results were obtained from crude Cox proportional hazards regression analyses and are presented as hazard ratios (HR) and 95% confidence intervals (CI) for one standard deviation change of serum GPC4. Abbreviations: BMI, body mass index; eGFR, estimated glomerular filtration rate; T2DM, type 2 diabetes mellitus; CAD, coronary artery disease; ACE, angiotensin converting enzyme.

**Table 5:** Association between serum GPC4 and all-cause mortality in subgroup analyses.

	N° of controls/events	HR [95%CI]	*p-value*	*P*_interaction_
Age < 67 years	340/34	1.51 [1.24–1.83]	< 0.001	0.791
Age ≥ 67 years	275/111	1.56 [1.34–1.82]	< 0.001	
Male	376/104	1.56 [1.40–1.74]	< 0.001	0.935
Female	239/41	1.58 [1.24–2.03]	< 0.001	
BMI < 30 kg/m2	445/112	1.58 [1.42–1.76]	< 0.001	0.715
BMI ≥ 30 kg/m2	170/33	1.49 [1.13–1.97]	0.005	
eGFR ≥ 60 ml/min/1.73 m2	571/108	1.71 [1.43–2.05]	< 0.001	0.006
eGFR < 60 ml/min/1.73 m2	44/37	1.16 [0.93–1.43]	0.185	
No T2DM	477/80	1.61 [1.42–1.84]	< 0.001	0.213
T2DM	137/64	1.39 [1.15–1.68]	0.001	
No significant CAD	298/44	1.57 [1.24–1.84]	< 0.001	0.764
Significant CAD	317/101	1.64 [1.41–1.92]	< 0.001	
No statin treatment	303/77	1.58 [1.40–1.78]	< 0.001	0.707
Statin treatment	312/68	1.51 [1.24–1.84]	< 0.001	
No beta blocker treatment	272/63	1.46 [1.26–1.68]	< 0.001	0.035
Beta blocker treatment	343/82	1.88 [1.56–2.27]	< 0.001	
No ACE inhibitor treatment	443/88	1.49 [1.30–1.70]	< 0.001	0.060
ACE inhibitor treatment	172/57	1.92 [1.55–2.37]	< 0.001	

The results were obtained from crude Cox proportional hazards regression analyses and are presented as hazard ratios (HR) and 95% confidence intervals (CI) for one standard deviation change of serum GPC4. Abbreviations: BMI, body mass index; eGFR, estimated glomerular filtration rate; T2DM, type 2 diabetes mellitus; CAD, coronary artery disease; ACE, angiotensin converting enzyme.

## 2 Experimental Design, Materials and Methods

Our study included 760 patients who underwent elective coronary angiography to assess the status of suspected or known CAD at the Academic Teaching Hospital Feldkirch, Austria [1]. Coronary angiography was performed using the standard Judkin's technique. Significant CAD was defined as ≥ 50% lumen narrowing of coronary artery stenoses [2]. Diagnosis of type 2 diabetes mellitus (T2DM) was made using the definition of the American Diabetes Association (ADA) [3]. Laboratory measurements were performed on sera of venous blood samples collected after a 12 h overnight fasting period. The ‘Chronic Kidney Disease Epidemiology Collaboration’ (CKD-EPI) creatinine equation was applied to estimate the glomerular filtration rate (eGFR) [4]. Serum GPC4 levels were determined using a commercially available ELISA kit (Cloude-Clone, Houston, Texas) according to the manufacturer's instructions.

Over a mean follow up period of 6.3 ± 1.8 years the incidence of MACE, vascular mortality, and all-cause mortality were documented. MACE was defined as the composite of nonfatal myocardial infarction, nonfatal stroke, and vascular mortality. Vascular mortality includes mortality from congestive heart failure due to CAD, death from myocardial infarction, fatal ischemic stroke, or sudden cardiac death. Date and causes of death were obtained from a national survey (Statistik Austria, Vienna, Austria) or from hospital records.

Differences between GPC4 levels and categorical variables were tested for statistical significance using the Mann–Whitney–U test. Hazard ratios (HRs) along with their 95% confidence intervals (CIs) for the incidence of first events were generated using Cox proportional hazards regression. Continuous variables were z-transformed for these calculations. The optimal threshold values of GPC4 for predicting MACE, vascular mortality, and all-cause mortality were assessed using receiver operating characteristic curve (ROC) analysis and the Youden's index method [5]. In order to evaluate the value of GPC4 as a predictive biomarker, Cox regression models were fitted with the respective study endpoint as the dependent variable and C-statistics were applied. The basic risk models comprised sex, age, T2DM, smoking, BMI, total cholesterol, C-reactive protein, blood pressure, eGFR, and angiographically determined significant CAD as independent variables. The models were compared to further models including as additional marker GPC4. The predictive power of the regression models were measured by calculation of Harrell's C and Somers' D. Furthermore, we determined time-dependent ROC curves and the respective area under the curve applying nearest neighbor estimation method. SPSS 26.0 (SPSS, Inc., Chicago, IL) and R statistical software v. 3.2.3 (http://www.r-project.org) were used for statistical analyses.

## Ethics Statements

We hereby state that the research has been carried out in accordance with The Code of Ethics of the World Medical Association (Declaration of Helsinki), that the Ethics Committee of the University of Innsbruck approved this study (EK-2-2008/0017) and that all participants gave written informed consent.

## CRediT authorship contribution statement

**Axel Muendlein:** Conceptualization, Methodology, Formal analysis, Writing – original draft, Supervision. **Eva Maria Brandtner:** Methodology, Investigation, Validation, Writing – review & editing. **Andreas Leiherer:** Validation, Formal analysis, Writing – review & editing. **Kathrin Geiger:** Investigation, Writing – review & editing. **Christine Heinzle:** Investigation, Writing – review & editing. **Stella Gaenger:** Investigation, Writing – review & editing. **Peter Fraunberger:** Methodology, Validation, Writing – review & editing. **Arthur Mader:** Validation, Writing – review & editing. **Christoph H. Saely:** Supervision, Writing – review & editing. **Heinz Drexel:** Conceptualization, Writing – review & editing, Supervision, Project administration.

## Declaration of Competing Interest

The authors declare that they have no known competing financial interests or personal relationships that could have appeared to influence the work reported in this paper.

## Data Availability

Data on the association of serum glypican-4 with future major adverse cardiovascular events and mortality in patients undergoing coronary angiography (Original data) (Mendeley Data).
